# A computational model of posterior parietal circuits during decision making and sequential planning

**DOI:** 10.1186/1471-2202-13-S1-P159

**Published:** 2012-07-16

**Authors:** Yuhui Li, He Cui

**Affiliations:** 1 Brain and Behavior Discovery Institute, Georgia Health Sciences University, Augusta, GA 30912, USA; 2 Department of Psychiatry and Health Behavior, Georgia Health Sciences University, Augusta, GA 30912, USA

## 

In the posterior parietal cortex (PPC), three anatomically separated but reciprocally interconnected subareas, the lateral intraparietal area (LIP), parietal reach region (PRR) and dorsal area 5 (area 5d), are involved in sensorimotor transformation during planning of eye/arm movements. We have previously designed the saccade/reach effector choice and sequential reach paradigms to dissociate cognitive and executive planning activity in PPC. During saccade/reach choice, LIP and PRR encodes potential saccade/reach plans in parallel before effector decision is formed, and the selected saccade/reach plans thereafter [1], whereas area 5d only reflects the decision outcome [2]. During sequential reach, PRR encodes both immediate and subsequent goals [3], whereas area 5d only represents the immediate upcoming reach [4].

Based on the experimental results above, we propose a network model to conceive the functional connectivity across these areas. The model is composed of an up-stream layer representing the abstract motor intentions and a down-stream layer representing concrete motor commands. The up-stream layer consists of LIP and PRR neurons preferring different directions for saccade and reach, respectively, and two populations compete against each other. The down-stream layer is composed of area 5d neurons preferring arm movements to different directions. In each neuronal pool, each leaky integrate-and-fire neuron excites others representing nearby targets but inhibits the remaining. The up-stream layer also feed-forwards to the down-stream layer to excite the neurons preferring the same effector and nearby target but to inhibit others. Furthermore, each LIP and PRR neuron receives self-excitation whose magnitudes are modulated by the rule-based behavioral context, reflecting the top-down feedback.

Our simulation results show that, during the effector choice and instructed tasks, the effector information is ambiguous at first so that both LIP and PRR show strong responses and counterbalance the total input to area 5d. Once effector information is determined by either choice or instruction, LIP and PRR activities differentiate to trigger or suppress area 5d activity. During the classical memory saccade/reach tasks, effector information is given at the cue onset so that LIP and PRR activities began to differentiate and area 5d fires immediately. During the sequential reaching task, after two targets are simultaneously presented, the activity of the PRR encoding the 1st goal increases faster while the activity of the PRR encoding the 2nd goal increases slower but lasts longer due to the different recurrent connections modulated by the behavioral context. Consequentially, only the activity of the area 5d neuron encoding the 1st goal is elevated before the GO signal. Once the first reach is completed, the area 5d neuron preferring the 2nd goal begins to fire because top-down context information (efference copy in this case) reduces the strength of recurrent connection for the PRR neuron coding the 1st goal.

Preliminary results indicate that our model captures all characteristics of neural activity we have observed so far. Our ongoing modeling work will help reveal the network mechanisms underlying the operation of the parietal sensorimotor circuits.
